# The big five model in bipolar disorder: a latent profile analysis and its impact on longterm illness severity

**DOI:** 10.1186/s40345-021-00248-y

**Published:** 2022-01-18

**Authors:** Niklas Ortelbach, Jonas Rote, Alice Mai Ly Dingelstadt, Anna Stolzenburg, Cornelia Koenig, Grace O’Malley, Esther Quinlivan, Jana Fiebig, Steffi Pfeiffer, Barbara König, Christian Simhandl, Michael Bauer, Andrea Pfennig, Thomas J. Stamm

**Affiliations:** 1 Department of Educational Science and Psychology, Free University of Berlin, Berlin, Germany; 2 Department of Psychiatry and Psychotherapy, Charité – Universitätsmedizin Berlin, Berlin, Germany; 3 Department of Psychology, Brandenburg Medical School Theodor Fontane, Fehrbelliner Straße 38, 16816 Neuruppin, Germany; 4 Department of Pediatrics, Division of Oncology and Hematology, Charité – Universitätsmedizin Berlin, Berlin, Germany; 5 Department of Psychiatry and Psychotherapy, Carl Gustav Carus University Hospital, Technische Universität Dresden, Dresden, Germany; 6 Bipolar Center, Wiener Neustadt, Austria; 7 Faculty of Medicine, Sigmund Freud University Vienna, Vienna, Austria; 8 Schloss Luetgenhof Hospital, Centre for Personal Medicine, Psychosomatics and Psychotherapy, Dassow, Mecklenburg-Western Pomerania Germany

**Keywords:** Bipolar disorder, Big five, Personality typology, Morbidity index, Illness course

## Abstract

**Background:**

Using a personality typing approach, we investigated the relationship between personality profiles and the prediction of longterm illness severity in patients with bipolar disorder (BD). While previous research suggests associations between BD and traits from the NEO-FFI profiles, the current study firstly aimed to identify latent classes of NEO-FFI profiles, and, secondly, to examine their impact on the longterm prognosis of BD.

**Methods:**

Based on the NEO-FFI profiles of 134 euthymic patients diagnosed with BD (64.2% female, mean age = 44.3 years), successive latent profile analyses were conducted. Subsequently, a subsample (n = 80) was examined prospectively by performing multiple regression analysis of the latent classes to evaluate the longitudinal course of the disease (mean: 54.7 weeks) measured using a modified Morbidity Index.

**Results:**

The latent profile analyses suggested a 3-class model typifying in a resilient (n = 68, 51%), vulnerable (n = 55, 41%) and highly vulnerable (n = 11, 8%) class. In the regression analysis, higher vulnerability predicted a higher longterm Morbidity Index (*R*^2^ = 0.28).

**Conclusions:**

Subgroups of patients with BD share a number of discrete personality features and their illness is characterized by a similar clinical course. This knowledge is valuable in a variety of clinical contexts including early detection, intervention planning and treatment process.

## Introduction

The present study uses the *Big Five* personality typology to predict the longterm course of bipolar disorder (BD), thus addressing the gap in prospective research of this recurring psychiatric disorder. There is an ongoing debate about how personality factors relate to the development, expression and prognosis of clinical disorders. Klein, Durbin (Klein et al. 2009) illustrated six models to describe the nature of the association between personality and mood disorders. The *common cause* and *precursor* models view personality as an antecedent to the disorder, where both are underpinned by the same etiological factors. In the precursor model, personality traits represent early, subclinical features of the disorder. The *predisposition* and *pathoplasticity* models depict personality as a risk factor for the onset (or severity and course, respectively) of a disorder without implying the same etiological origin. With a reverse causal direction, the *concomitant (state-dependent)* model assumes that personality assessments are influenced by the patient’s current mood state. In a similar vein, the *complication (scar)* model proposes that personality is permanently altered by the disorder with no return to its baseline state.

The *Big Five* personality comprises independent traits of neuroticism, extraversion, openness to experience, agreeableness and conscientiousness (McCrae and John 1992) and forms the basis of several personality inventories (Costa and McCrae 1992). Subjects may not only be differentiated by their score on individual personality factors, but also on *profiles* composed of these factors. Thus, subgroups (or classes) of similar profiles can be identified. Latent profile analysis (Muthén and Muthén 2015) is an extension of latent class analysis assuming unobserved (latent) categorical variables (classes) to explain similarities between continuous manifest indicators. For example, in a therapy study with major depressive disorder patients, (Wardenaar et al. 2014) extracted two latent classes based on the *NEO Five-Factor Inventory* (NEO-FFI; German version: (Borkenau and Ostendorf 2008)): The vulnerable class was characterized by high neuroticism as well as low extraversion and conscientiousness, while the resilient class was characterized by moderate neuroticism and extraversion, and high agreeableness and conscientiousness. Furthermore, the authors showed that belonging to the resilient personality class was predictive of being successfully treated more quickly.

Bipolar affective disorders are chronic psychiatric conditions characterized by recurring depressive and manic (bipolar type I) or hypomanic (bipolar type II) mood episodes, with inter-episode intervals of remission or decreased symptoms (euthymia). Certain personality factors have already been linked with particular psychiatric disorders (Malouff et al. 2005), however a line of research has extended on this topic by exploring personality variables as possible predictors of the clinical course of BD. For example, Lozano and Johnson (Lozano and Johnson 2001) found that high neuroticism longitudinally predicted increasing depressive symptoms, while high conscientiousness predicted increasing manic symptoms. Further, neuroticism has been found to predict poorer sleep quality (Saunders et al. 2013). Neuroticism, agreeableness, and conscientiousness affect suicidality (Aaltonen et al. 2016), and low extraversion, openness and conscientiousness are associated with polypharmacy (Sachs et al. 2014). Previous research has also indicated several other demographic and clinical variables to be related to the course of BD. These include gender, age at onset, pattern of episodes, BD subtype, rapid cycling, depressive symptoms, and co-morbid conditions (Saunders and Goodwin 2018; Treuer and Tohen 2010). Additionally, the development of BD from an initial unipolar depression diagnosis was associated with a higher level of extraversion (Bukh et al. 2016).

In studies where the course of BD has been measured prospectively, *period until next affective episode* (time to relapse) has generally been regarded as an essential outcome measure. However, Baethge et al*.* (Baethge et al. 2003) have argued for a more sophisticated approach, namely using the Morbidity Index (Coppen et al. 1976). The MI is calculated based on weekly observations of the patient’s symptomatology while controlling for total observation time. By continuously collecting data, a more robust measure of the course of the illness is achieved. In addition to this concept, we introduced also subsyndromal symptoms into the calculation of the MI (Rote et al. 2018; Bonnin et al. 2012) as there is much evidence that bipolar patients also can be differentiated by a strict euthymia criteria versus subsyndromal depressive or hypomanic symptoms.

With the present study being one of the first of its kind to examine bipolar patients' personality profiles as prospective course modifiers, the general goal of the research is to provide a basis for using a comprehensible typology in communication with clinical professionals or patients. Such a typology would be invaluable to clinicians in screening for early BD symptoms; for deriving appropriate, individually-tailored interventions; and for establishing the prognosis and probable treatment outcome. The aims of this study are therefore twofold: First, we aim to classify BD based on patients' NEO-FFI scores by applying latent profile analysis and to characterize the emerging latent classes. In a second step, we aim to predict the treatment course (indicated by the MI) using the obtained personality classes.

## Method

### Participants

The sample consisted of 134 patients (64.2% female, mean age = 44.3 years), 64.9% of whom had been diagnosed with bipolar I disorder while the remaining 29.9% had a bipolar II diagnosis. Further demographic features and disorder-related characteristics are listed in Table 1 (see column *Total sample*).

**Table 1:** Sample characteristics and differences between personality classes

Variable	Total sample (N = 134)	Class 1: resilient (n = 68)	Class 2: vulnerable (n = 55)	Class 3: highly vulnerable (n = 11)	Statistics	
					p value	Effect size
Bipolar I subtype (% yes)	87 (64.9)	47 (69.1)	34 (61.8)	6 (54.5)	0.675^a^	*V* = 0.07^b^
Demographic variables						
Gender (% female)	86 (64.2)	43 (63.2)	35 (63.6)	8 (72.7)	0.892^a^	*V* = 0.05^b^
Years of age—*M* (*SD*)	44.3 (13.3)	45.1 (13.5)	43.7 (13.5)	41.9 (10.7)	0.705^c^	ω^2^ = −0.01
# Years of education—*M* (*SD*)	13.5 (3.2)	13.8 (3.1)	13.4 (3.3)	12.2 (2.8)	0.262^d^	η^2^ = 0.01
Employment (% yes)	69 (51.5)	34 (50.0)	30 (54.5)	5 (45.5)	0.797^a^	*V* = 0.06^b^
Relationship Status (% in relationship)	69 (51.5)	37 (54.4)	26 (47.3)	6 (54.5)	0.755^a^	*V* = 0.07^b^
Clinical variables						
Substance use (% yes)						
Alcohol	39 (29.1)	21 (30.9)	15 (27.3)	3 (27.3)	1.00^a^	*V* = .03^b^
Drugs	16 (11.9)	8 (11.8)	6 (10.9)	2 (18.2)	0.774^a^	*V* = 0.06^b^
Co-morbidity^e^—*M* (*SD*)	0.31 (0.64)	0.2 (0.52)	0.29 (0.52)	1.25 (1.16)	0.002** ^d^	η^2^ = 0.10
Medication^f^—*M* (*SD*)	1.80 (1.01)	1.78 (1.18)	1.78 (0.85)	2.1 (0.57)	0.326^d^	η^2^ = 0.00
Age at onset—*M* (*SD*)	27.2 (10.2)	29.3 (9.6)	24.5 (10.2)	27.3 (11.3)	0.023* ^d^	η^2^ = 0.04
Past episodes—*M* (*SD*)	15.4 (16.2)	14.9 (17.9)	16.3 (15.5)	13.6 (10.1)	0.425^d^	η^2^ = 0.00
Previous rapid cycling (% yes)	27 (20.1)	11 (16.2)	14 (25.5)	2 (18.2)	0.458^a^	*V* = 0.11^b^
#Hospitalizations—*M* (*SD*)	3.0 (3.1)	3.0 (2.6)	2.8 (3.4)	4.3 (3.8)	0.314^d^	η^2^ = 0.00
Attempted suicide (% yes)	41 (30.6)	19 (27.9)	20 (36.4)	2 (18.2)	0.478^a^	*V* = 0.12^b^
NEO-FFI scales—M (SD)						
Neuroticism	1.96 (0.71)	1.44 (0.45)	2.39 (0.42)	3.10 (0.34)	< 0.001*** ^d^	η^2^ = 0.67
Extraversion	1.99 (0.6)	2.25 (0.48)	1.88 (0.52)	1.0 (0.43)	< 0.001*** ^d^	η^2^ = 0.27
Openness	2.43 (0.55)	2.36 (0.59)	2.54 (0.46)	2.25 (0.55)	0.103^c^	ω^2^ = 0.02
Agreeableness	2.53 (0.43)	2.68 (0.38)	2.45 (0.41)	2.04 (0.43)	< 0.001*** ^c^	ω^2^ = 0.17
Conscientiousness	2.52 (0.55)	2.85 (0.4)	2.31 (0.41)	1.55 (0.25)	< 0.001*** ^c^	ω^2^ = 0.49
Psychopathology						
HDRS-21, *M (SD)*	2.92 (2.74)	1.85 (2.17)	3.82 (2.79)	4.50 (3.46)	< 0.001***	η^2^ = 0.12
YMRS, *M (SD)*	1.46 (2.21)	1.94 (2.74)	1.06 (1.49)	0.75 (1.39)	0.384	η^2^ = 0.00
Morbidity Index—*M* (*SD*)	0.44 (0.37)	0.30 (0.31)	0.53 (0.35)	0.9 (0.35)	< 0.001*** ^d^	η^2^ = 0.19

NEO-FFI, NEO Five-Factor Inventory; HDRS-21, Hamilton Depression Rating Scale-21; YMRS, Young Mania Rating Scale

^a^Fisher-Freeman-Halton exact test, ^b^Cramer’s *V*, ^c^F(2131), ANOVA, ^d^H(2), Kruskal–Wallis test, asymptotic significance, ^e^number of co-morbid axis I or II diagnoses, ^f^number of current psychotropic medication groups (lithium, anticonvulsants, neuroleptics, and antidepressants)

*p < 0.05, ** *p* < 0.01, *** *p* < 0.001

Participants were part of a larger, prospective multi-center study conducted between May 2004 and 2011 in three university clinics across Germany and Austria (Berlin, Dresden, and Neunkirchen). For the present analyses, subgroups that completed the NEO-FFI (NEO-FFI; German version: (Borkenau and Ostendorf 2008)) were examined.

Participants were recruited from outpatient clinics specialized in the treatment of BD. The following primary inclusion criteria applied: Diagnosis of BD I/II according to the *Structured Clinical Interview for DSM-IV* (SCID I/II; German version: (Wittchen et al. 1997)), treatment with psychopharmacological drugs, mental and physical capabilities that enabled the patient to participate and establish informed written consent (e.g., fluent German language). Exclusion criteria were having a diagnosis of an organic brain disorder, having another predominant axis I disorder, and acute suicidality.

## Measures

### Diagnoses and psychopathology

While patients were previously diagnosed with BD, their diagnosis was verified by clinical interview using the SCID. Current psychopathological state was assessed using the *Hamilton Depression Rating Scale* (HDRS-21, (Hamilton 1967)) and the *Young Mania Rating Scale* (YMRS, (Young et al. 1978)), both of which provided observer ratings on the severity of depressive and (hypo-)manic symptoms, respectively.

### Personality assessment

Participants chosen for the current analyses completed the *NEO-FFI* at baseline. This self-report instrument consists of 60 statements rated on a 5-point Likert-type scale (1 = *strongly disagree* to 5 = *strongly agree*). Its factor structure conforms to the five factor model of personality ((Rosellini and Brown 2011), for a clinical sample) and every subscale has shown adequate internal consistency and temporal stability (α = 0.72 to 0.87, r = 0.71 to 0.82, 6). Personality assessment was conducted only if patients were in stable remission.

### Prospective assessment

After baseline assessment, the patients’ course of illness was investigated prospectively at least every eight weeks in terms of the following variables: Current psychopathology (HDRS, YMRS), duration and polarity of relapse, change of medication, type of medication, and necessity of hospitalization. Longitudinal illness severity was measured using the Morbidity Index (MI), a measure adopted in a number of pharmacotherapy and treatment response studies on affective disorders (Baethge et al. 2003; Coppen et al. 1976). The MI provides information pertaining to both (a) duration of symptoms endured as well as (b) the severity of the period of illness and is therefore considered as a more refined measure than, for example, frequency of episodes or hospital admissions (Baethge et al. 2003; Rote et al. 2018). At each follow-up assessment, scores on the MI were updated based on the psychiatrist’s current psychopathology rating; where ‘degree 0’ indicated no symptoms, ‘degree 1’ signified mild symptoms, ‘degree 2’ signified aggravation with a need for additional treatment, and ‘degree 3’ signified aggravation and an acute need for hospitalization. In order to detect minor depressive and manic symptoms, the following modification was made to the MI based on Rote et al*.* (Rote et al. 2018): A subthreshold parameter of 0.5 was integrated using adjusted cutoff scores on symptom measures (HDRS-21 ≥ 4 and ≤ 9; YMRS ≥ 3 and ≤ 11) on the basis that the gap between degrees 0 and 1 can be considered too wide. The resulting sum was divided by the total observation time, resulting in values ranging between zero and 1.47 (*M* = 0.44, *SD* = 0.37), with a high MI indicating a poorer outcome.

### Procedures

**Fig. 1:**
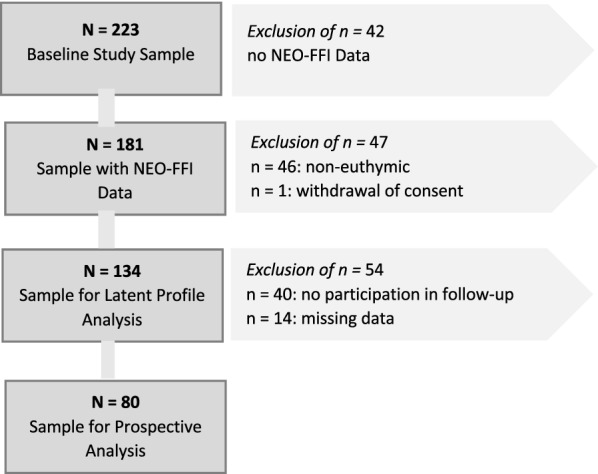
Flowchart of the sample including reasons for the exclusion of patients. NEO-FFI (NEO Five-Factor Inventory)

Figure 1 displays the exclusion of patients across different stages of our investigation and the resulting sample sizes for the respective analyses.

Altogether, the baseline assessment involved extensive collection of demographic and psychological variables (personality, quality of life, sleep quality), psychopathology (clinical and self-ratings of depression and [hypo-]mania), anamnestic information (patient history concerning past episodes, rapid cycling, hospitalization, suicide attempts, substance consumption) as well as past and current medication. To minimize the potential risk of assessing concomitant current mood states, the personality assessment was only proceeded providing those participants were euthymic; which was confirmed by assessment using the SCID interview and other symptom measures (HDRS-21 ≤ 9 and YMRS ≤ 12). If euthymia criteria were not met, participants were excluded.

The follow-up sessions were scheduled every eight weeks for 2 years, but actual observation time varied among patients due to various reasons, e.g., illness or hospitalization (Range = 1 to 122 weeks, *M* = 54.7, *SD* = 34.9). Where assessment intervals exceeded 20 weeks, or where total observation time was less than eight weeks, participants were omitted from further analyses. This resulted in a final sample of 94 patients who provided prospective data, of whom 80 yielded valid data on the selected predictor variables and were thus used in the multiple regression analysis.

### Statistical analyses

Means for the five NEO-FFI subscales were calculated based on the valid items (ipsative mean imputation; (Schafer and Graham 2002)) taking into account the low rate of missing data (0.8% of the values) and the reliability of the NEO-FFI scales. We evaluated the pattern of missing data among the demographic and clinical variables by applying Little’s (Little 1988) omnibus MCAR test which indicated that data were missing due to completely random purposes; Χ^2^(349) = 340.57, *p* = 0.617. Hence, we applied pairwise deletion of the affected cases to test for group differences.

*Latent profile analysis* (Muthén and Muthén 2015) was used to group similar patients using probability based on their individual NEO-FFI profiles. For our analyses, all five NEO-FFI subscales were entered into the latent class model. We chose maximum likelihood estimation with robust standard errors, with variances held equal across classes and covariance among the latent class indicators fixed at zero. To reduce the risk of local maxima, the initial number of random starting value sets was set at 500, and the 50 best sets (according to their likelihood values) were selected for optimization after 50 iterations (Geiser 2012). After conducting a series of successive latent profile analysis models with increasing numbers of latent classes, the decision regarding the number of obtained classes was empirically and conceptually driven (i.e., being mindful of the interpretability of the class solution).

To validate the latent classes, *group comparisons* were performed to test for differences at baseline. Analyses of variance (ANOVA) were performed for parametric variables. Kruskal–Wallis tests and subsequent Man-Whitney tests with Bonferroni corrections for multiple post hoc comparisons were used for non-parametric variables. Dichotomous categorical variables were analyzed using Fisher’s exact test.

To assess the longitudinal treatment effect, a *hierarchical multiple regression* was conducted with the MI as the outcome variable, regressed on the dummy-coded latent classes as well as on a set of clinical variables previously found to predict the course of BD (Saunders and Goodwin 2018). Of the potential predictor variables, those with significant correlations with either the outcome or another chosen predictor were included in the regression analysis (Cohen et al. 2003).

We used Mplus version 7.4 (Muthén and Muthén 2015) to perform the latent profile analyses, and IBM SPSS Statistics version 28.0 for all further analyses.

## Results

### Clinical characteristics

Table 1 (column *Total Sample*) summarizes the clinical features of our sample. With an average age at onset of 27.2, our subjects had an average of 15.4 past episodes during their course of BD and a mean of three hospitalizations. Twenty percent suffered from previous rapid cycling and 30.6% reported previous suicide attempts.

### Latent profile analysis

With regards to our primary research question, the detailed results for the increasing latent class models are displayed in Table 2. We selected the 3-class model to represent our data as it achieved the lowest BIC value which is recommended when determining the number of latent classes (Nylund et al. 2007). In comparison to the 2-class model, the Bootstrap Likelihood Ratio Test and Vuong-Lo-Mendell-Rubin Likelihood Ratio Test both indicated a better fit for the 3-class model, which, furthermore, exhibited a higher entropy value.

**Table 2:** Latent class model fit indices

Model	FP	Log-likelihood	Entropy	AIC	BIC	aBIC	BLRT *p* value	VLMRT *p* value
1-class	10	− 561.1	–	1142.1	1171.1	1139.5	–	–
2-class	16	− 525.3	0.687	1082.6	1128.9	1078.3	0.000***	0.020*
**3-class**	**22**	− **509.9**	**0.750**	**1063.7**	**1127.5**	**1057.9**	0.000***	0.009**
4-class	28	− 501.4	0.750	1058.7	1139.9	1051.3	0.112	0.337
5-class	34	− 493.5	0.785	1055.0	1153.5	1046.0	0.208	0.509
6-class	40	− 487.4	0.812	1054.9	1170.8	1044.3	0.598	0.394

The best fit model is shown in bold. FP, number of free parameters; AIC, Akaike information criterion; BIC, Bayesian information criterion; aBIC, sample size-adjusted BIC; BLRT, Bootstrap Likelihood Ratio Test *p* value for (k−1) classes; VLMRT, Vuong-Lo-Mendell-Rubin Likelihood Ratio Test *p* value for (k−1) classes

* *p* < .05, ** *p* < .01, *** *p* < .001

**Fig. 2:**
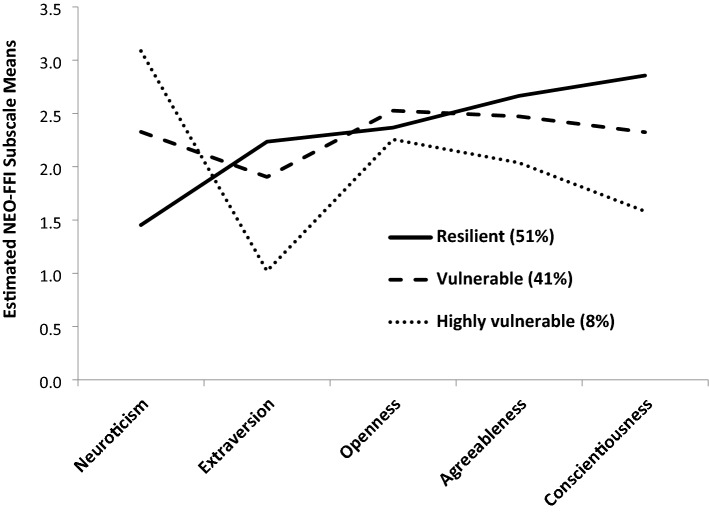
Class-specific personality profiles for the 3-class model. NEO-FFI, NEO Five-Factor Inventory

Figure 2 displays the plotted NEO-FFI subscale means of the three classes based on the estimated model. The resulting classes were characterized by their low/high scores on the Big Five scales in comparison to the means of the other classes. We interpreted and labeled the classes according to (Wardenaar et al. 2014) as *resilient, vulnerable* and *highly vulnerable,* whereby:

- *Resilient* class (n = 68; or 51% of our sample) is characterized by low neuroticism, high extraversion, high agreeableness and high conscientiousness,
- *Vulnerable* class (n = 55; 41%) is characterized by higher neuroticism, lower extraversion, lower agreeableness and lower conscientiousness, and
- *Highly vulnerable* class (n = 11; 8%) is characterized similar to the *vulnerable* class but with even higher neuroticism, lower extraversion, lower agreeableness and lower conscientiousness.

### Personality class characteristics

To validate our latent class model, we examined descriptive characteristics of the obtained three classes. Results of these secondary explorative analyses are shown in Table 1. The classes did not differ in terms of any *socio-demographic variable*. To address the question as to which clinical variables might be associated with belonging to a specific personality class, the analyses revealed the number of co-morbid diagnoses and age at onset as significant correlates. Pair-wise comparisons regarding the former indicated significantly more *co-morbid disorders* in the highly vulnerable (*Mdn* = 1.5) compared to the vulnerable (*Mdn* = 0) and resilient (*Mdn* = 0) classes (*U* = 79, *p* = 0.010, *r* = 0.38 and *U* = 106, *p* < 0.001, *r* = 0.43, respectively). The resilient and vulnerable classes did not differ in their number of co-morbid disorders (*U* = 947, *p* = 0.188, *r* = 0.14). With regard to *age at onset,* pair-wise comparisons of the obtained classes indicated a significant difference between the vulnerable and resilient classes (*U* = 1280, *p* = 0.006, *r* = 0.25) with patients in the vulnerable class having an earlier onset age (*Mdn* = 21.5) than those in the resilient class (*Mdn* = 29). Age at onset in the highly vulnerable class did not significantly differ from the other classes (*Mdn* = 27, *U* = 321, *p* = 0.495, *r* = 0.08 and *U* = 256.5, *p* = 0.478, *r* = 0.09, respectively).

### Prediction of longterm illness course

To answer our second research question regarding the influence of our identified personality classes on the illness course, we initially scanned the correlation matrix of potential predictors and the MI. Significant correlates of the MI were the number of past episodes, *r*_S_ = 0.24, p = 0.028, and assignment to the latent personality class, *r*_S_ = 0.44, p < 0.001. Furthermore, the number of past episodes was correlated with rapid cycling (lifetime), *r*_S_ = 0.49, p < 0.001, and the personality class variable correlated with age at onset, *r*_S_ = -0.21, p = 0.040, and the number of co-morbid diagnoses, *r*_S_ = 0.27, p = 0.010. Due to parsimony of the intended regression model (Cohen et al. 2003), we did not inspect further significant correlates of potential predictor variables.

Table 3 displays the results of the multiple regression analyses predicting patient MI. In a first step, we simultaneously entered the number of co-morbid diagnoses, age at onset, number of past episodes, and rapid cycling into the regression model. None of these variables significantly predicted our outcome, *R*^2^ = 0.11, *F*(4,75) = 2.28, *p* = 0.069. In a second step, we added the dummy-coded personality classes. This resulted in a significant improvement predicting the MI, *R*^2^ = 0.27, Δ*R*^2^ = 0.16, *F*(6,73) = 4.53, *p* < 0.001. Thus, the final model adequately represented our data. In the final model, the only significant predictors were the two personality class variables (β = 0.31 and 0.39, *p’*s = 0.004 and 0.001, respectively).

**Table 3:** Results of the multiple regression analysis (with Morbidity Index as the unit of outcome, n = 80)

	*B* (*SE*)	β	*t*	*p*	95% confidence interval for *B*
Final regression model					
Constant	0.250 (0.125)		1.994	0.050	[0.000, 0.500]
Vulnerable vs. resilient class	0.234 (0.079)	0.312	2.956	0.004**	[0.076, 0.392]
Highly vulnerable vs. resilient class	0.538 (0.159)	0.385	3.390	0.001**	[0.222, 0.854]
Age at Onset	− 0.002 (0.004)	− 0.062	− 0.570	0.571	[− 0.010, 0.005]
Past episodes	0.004 (0.002)	0.208	1.702	0.093	[− 0.001, 0.009]
Co-morbidity	0.024 (0.061)	0.044	0.390	0.698	[− 0.098, 0.145]
Previous rapid cycling	0.024 (0.108)	0.028	0.222	0.825	[− 0.191, 0.239]

*R*^2^ = 0.108 for Step 1 (not shown), Δ*R*^2^ = 0.163 for Step 2 (*p* < 0.001)

** *p* < 0.01

To evaluate the accuracy of the regression model we examined residual and influential statistics. Five cases (6.25%) had absolute studentized residuals > 1.96 indicating potential influence on the regression model, but none had a Cook’s distance > 1. No case had an absolute standardized DFBeta > 2, thus indicating no substantial influence on the regression parameters (Stevens 2009). In order to assess the generalizability of our regression model, we calculated an adjusted *R*^2^ of 0.13 using Stein’s formula (Stevens 2009) and assessed several regression assumptions. The Durbin-Watson statistic of *d* = 1.96 suggested uncorrelated residuals, and a non-significant Kolmogorov–Smirnov test indicated normal distribution of the standardized residuals, *D*(80) = 0.07, *p* = 0.20. All tolerance values were > 0.2 implying no potential collinearity problems between the considered predictors, although the average variance inflation factor (VIF) was 1.33.

## Discussion

### Summary of the results

This study is the first prospective investigation to show the impact of personality profiles on the course of BD. We successfully identified subgroups of BD patients based on their NEO-FFI profiles. The resulting 3-class model both represents the sample and sufficiently classifies patient groups. In addition, we observed that a poorer longitudinal outcome based on the MI was associated with belonging to either the vulnerable or highly vulnerable class. Personality classes accounted for 27% (adjusted *R*^2^ = 0.13) of the variation of the MI.

### Discussion of the obtained class solution

Our latent class solution extended upon Wardenaar et al*.*’s (Wardenaar et al. 2014) study with patients with major depressive disorder by identifying *resilient* and *vulnerable* classes, but also a third, *highly vulnerable* class. Despite methodical similarities, the novelty of the current study findings in a BD sample is noteworthy; particularly given the differences in the personality profiles of unipolar depression versus BD (Akiskal et al. 2006; Araujo et al. 2016). In terms of the obtained class solution, a key point to consider is the definite rejection of the 1-class model, hence, this finding strongly supports the search for subgroups in the observed sample of patients. Interestingly, openness to experience was the only personality dimension whereby none of the three detected classes differed. This may be interpreted in line with previous research, which has described openness as a distinct characteristic in patients with BD (Middeldorp et al. 2011; Nowakowska et al. 2005), whereas neuroticism and extraversion for example seem to be nonspecifically related to psychological disorders in general (Malouff et al. 2005).

Previous studies focusing on the link between BD and personality features were mostly limited to the comparison of euthymic patients with healthy control subjects or population norms. While there have been some relatively consistent findings—for example of elevated levels of neuroticism and lower levels of conscientiousness in BD (Middeldorp et al. 2011; Nowakowska et al. 2005)—there is notable disparity in the body of empirical studies. Our study addressed the discrete question as to whether this may be solely due to study characteristics, or rather alternatively, if the homogeneity of BD patients may in fact be a false assumption. Indeed, when BD subgroups have been described in previous studies, some differences emerged: For example, higher neuroticism scores were reported in BD type II vs. type I patients (Akiskal et al. 2006; Kim et al. 2012).

To validate our three personality classes, we searched for potential associations across a set of clinical variables. Our finding of an association between higher vulnerability and more co-morbid disorders is in line with previous research explaining co-morbidity by neuroticism and conscientiousness (Khan et al. 2005; Spinhoven et al. 2009). Both factors are essential in differentiating our highly vulnerable and resilient class. However, a considerable strength of our naturalistic study is its representativeness: Due to the inclusion of patients with co-morbid diagnoses, the results can be generalized to the majority of BD patients who indeed suffer from at least one additional axis I or II diagnosis (Bauer et al. 2005; McElroy et al. 2001). In line with the conclusion of (Leboyer et al. 2005) who linked earlier onset age of BD with more severe clinical features and a worse longterm outcome, patients in our vulnerable class reported an earlier onset age compared to the resilient class. The lack of observed difference in age at onset for the highly vulnerable class was not expected but may be explained by the small size of this subgroup in our study (n = 8).

### Discussion of the prospective results: morbidity index in BD

The second major finding of our study was that the MI provided crucial clinical information in measuring the prospective illness course and differentiated the three personality classes. The generalizability of our regression model is particularly important if we are to draw conclusions about BD populations beyond the current study’s sample; such as for instance the expectation of a poorer treatment outcome for patients with a highly vulnerable personality. The predictive value of the personality variables may be explained by several factors. First, previous research has linked personality—in particular agreeableness—and treatment outcome by means of its effects on the therapeutic alliance (Kushner et al. 2016). In this way, certain combinations of specific traits may serve as an asset or obstacle to the therapeutic intervention. According to (Miller 1991), high neuroticism indicating emotional instability may underlie an instable, poorer clinical course, with destabilizing effects in stressful situations or phases of life. Low extraversion may lead to withdrawing from or having reservations in relationships, thus results in the absence of social support which in turn triggers or maintains depression; while high extraversion conversely may be associated with positive coping behavior (Coulston et al. 2013). It is plausible that low conscientiousness may be related to a lack of investment in treatment.

While the utilization of the MI as an indicator of prospective illness course is still a relatively novel approach, it is advantageous over many of the most widely-used single measures (e.g., number of affective episodes, hospitalizations, time to remission or time to relapse). The MI considers both the duration of an affective illness episode, as well as the severity of depressive and/or (hypo-)manic symptoms. Our modified MI additionally integrates subthreshold symptomatology, and thus considers impairments that are relatively unique or specific to BD beyond acute psychopathology (Rote et al. 2018). The use of the MI might serve as a core asset in predicting clinical courses of BD by personality aspects where past studies using single measures failed to do so (e.g., (Sparding et al. 2017)).

### Methodical considerations & limitations of the study

Some particular limitations should be considered in interpreting the study’s findings. The relatively small sample size and the size of the third class in the model produced should be kept in mind. However, this group was retained as our subsequent analyses showed a unique set of clinical correlates, emphasizing the conceptual gain of considering the highly vulnerable class. It is possible that selection bias may have occurred as patients were recruited in a university hospital setting. However, likely due to high visibility and reach, the clinics in question received and accepted a wide range of referrals, ranging from self-referrals in early intervention and differential diagnosis, to more severe, treatment-resistant cases. Notably, our sample included only euthymic patients: We excluded over 40 participants to minimize the risk of current mood states as possible confounders for personality scores. The five factors of the NEO-FFI investigate personality features on a very broad and abstract level. Without information on the lower facet levels, clinical interpretations drawn could be considered vague and thus less meaningful. Conversely, we found several clinical variables related to the detected NEO-FFI-based latent classes.

The cross-sectional nature of our personality assessment limits the interpretability of the findings in terms of interdependency with the illness course. They may be interpreted in accordance to the *pathoplasticity* model as described by Klein et al. (Klein et al. 2009), where belonging to a more vulnerable class indicates a risk factor for a more severe and poorer course of bipolar disorder. However, pre-morbid data or personality assessment later in the course of the illness are lacking here, and thus conclusive arguments against the *common cause* or *complication model* cannot be drawn. To minimize *state-dependent influences* of psychopathology on personality assessment we included only euthymic patients in our analyses, given the evidence that personality assessments are biased by current mood state in depressive patients (Ormel et al. 2004; Sauer et al. 1997). Our classes still significantly differed in their level of depressive symptoms, where higher Hamilton Depression ratings were associated with higher vulnerability. Thus, potential subclinical depressive mood confounds cannot be eliminated. Interestingly, Wardenaar et al*.* (Wardenaar et al. 2014) found similar latent personality profiles in patients suffering from acute major depressive episodes which may indicate a psychopathological influence rather on the profile level than on the profile shape. Our regression model showed that, in contrast to the personality class variables, clinical variables did not predict the illness course. While there was notable variability in the patient observation times in our study, studies following BD patients for up to a period of 2 years such as in the current study are rare. It is recommended that future researchers employ longitudinal designs in order to assess the potential interdependencies of personality features and BD. Further, future researchers may consider profiling BD across the Ashton and Lee HEXACO six factor model (Ashton and Lee 2008), as this would offer interesting insights into the potential role of their proposed sixth factor, Honesty-Humility.

## Clinical implications & conclusion

A key strength of the presented approach is the interpretability of a single typology over and above the consideration of several independent personality dimensions. This approach arguably has the potential to simplify communication between professionals supporting patients in clinical care. Indeed, it also has the scope to improve communication between patients and caregivers in the context of providing psychoeducation. According to our results, it may be possible to anticipate the course of a patient’s illness based on individual personality features. Consequently, a brief personality screening may also become a heuristic part of the initial assessment process for the early detection of BD. This would reduce delays in diagnosis and, accordingly, reduce the likelihood of negative consequences associated with lack of detection, e.g., self-harm, co-morbidities, reduced ability to attain age-specific developmental tasks or inadequate treatment decisions (Conus et al. 2014), and overall illness burden.

Neuroticism is associated with mixed states (Koszewska and Rybakowski 2008) and can contribute to the experience of more adverse life events (Whittington and Huppert 1998). Ogrodniczuk et al. 2003 found extraversion, openness, conscientiousness and low neuroticism to predict a positive treatment outcome in group psychotherapy; a profile clearly akin to our resilient personality class. In contrast, specialized treatment has been found to result in quicker recovery in vulnerable—but not in resilient—patients (Wardenaar et al. 2014). Collectively, these findings indicate that patients with a resilient personality profile may be predestined for more efficient, low-threshold interventions (e.g., group therapy), while more vulnerable patients tend to profit from more intensive and individually-tailored treatments which are more effective at preventing the development of a more severe disease trajectory.

The extraction of latent personality classes in BD patients again underlines the heterogeneity of the disorder. Groups of patients in the bipolar spectrum sharing specific personality features are characterized by similar clinical courses. Despite its widespread utilization in personality psychology, the Five Factor Model still lacks findings in specific areas of clinical psychology. Particularly in the context of BD, empirical evidence is rare. Using a prospective study design and latent profile analysis, this study makes a valuable contribution by linking the personality research tradition of the five factor model to an applied clinical cohort, offering meaningful clinical implications regarding the prospective prediction of illness courses.

## Data Availability

The data sets generated and/or analysed during this study are not publicly available because participants did not agree to share their clinical data as an open source. They are, however, available from the corresponding author on reasonable request.

## Abbreviations

BD: Bipolar disorder
HDRS-21: Hamilton Depression Rating Scale—21 item version
MI: Morbidity index
NEO-FFI: NEO Five Factor Inventory
YMRS: Young Mania Rating Scale
